# Educational supportive group therapy and the quality of life of hemodialysis patients

**DOI:** 10.1186/s13030-020-00200-z

**Published:** 2020-10-15

**Authors:** Sajad Mansouri, Amir Jalali, Mahmoud Rahmati, Nader Salari

**Affiliations:** 1 Department of Geriatric and Psychiatric Nursing, School of Nursing and Midwifery, Kermanshah University of Medical Sciences, Kermanshah, Iran; 2 Substance Abuse Prevention Research Center, Research Institute for Health, Kermanshah University of Medical Sciences, Kermanshah, Iran; 3 Department of Biostatistics, Faculty of Public Health, Kermanshah University of Medical Sciences, Kermanshah, Iran

**Keywords:** Quality of life, ESRD, Group therapy

## Abstract

**Background:**

In addition to physical, mental, and social condition, ESRD and hemodialysis affect the quality of life of patients as well. Psychotherapy and non-pharmaceutical interventions are effective measures to add meaning to life, create a goal and motivation in life, and improve the quality of life in chronic patients. The effect of educational and supportive group therapy on the quality of life (QOL) of hemodialysis patients was examined.

**Methods:**

The study was carried out as an interventional quasi-experimental study with the participation of 64 patients who were selected through convenience sampling and based on the patient’s hemodialysis days (Saturday, Monday, and Wednesday patients as an experimental group and Sunday, Tuesday, and Thursday patients as a control group). There were 32 patients in each group. The experimental group received eight 50 min sessions including two sessions per week. The control group received the normal interventions. The participants were assessed using a demographics form and Kidney Disease Quality of Life Short Form before, immediately after, and 1 month after the intervention. The collected data was analyzed using SPSS (v.24).

**Results:**

The mean QOL scores of the experimental group before, immediately after, and 4 weeks after the intervention were 36.99, 43.3, and 44.9 respectively. Those of the control group were 36.39, 37.2, and 37.1 respectively. There was no significant difference between the two groups before the intervention (*P* > 0.05); however, the difference between the two groups was significant immediately after and 4 weeks after the intervention (*P* = 0.0001). The trend of score change in the experimental group was also significant (*p* < 0.05), and Tukey ad-hoc test showed significant differences between the scores before intervention and those immediately after and 4 weeks after the intervention (*p* < 0.05).

**Conclusion:**

In general, educational and supportive group therapy can expand the interpersonal relationships of hemodialysis patients and positively affect their quality of life.

## Introduction

The prevalence of end stage renal disease (ESRD) is growing along with population growth and the expansion of urban life [1]. In addition, chronic kidney disease (CKD) has become a serious public health issue due to the increase in the prevalence and mortality rate of the disease [2]. One of the main challenges of health systems in the twenty-first century is that there will be1,200 ESRD patients in each million population by 2020 [3]. The number of HD patients is increasing by 15% each year in Iran [4]. ESRD patients have three options, including hemodialysis, kidney transplantation, and peritoneal dialysis [5, 6]. Not all renal disease patients have the chance of kidney transplantation [7]. The most common form of dialysis is hemodialysis (HD) performed three times per week in a hemodialysis center [3, 8, 9].

Patients under HD have different physiological experiences, such as fatigue, limited physical activity, decreased blood pressure, muscle spasm, nausea and vomiting, limitations in doing normal activities, and interruption of everyday life [10]. In addition, HD creates problems such as limitations of consuming liquids and foods, physical activity limitations, performance disorders, therapeutic problems during a dialysis session, and career problems [5]. One-half of ESRD patients report chronic pain, fatigue, cognitive disorders, depression, and anxiety [11]. Studies have shown that 65% of renal patients under HD suffer at least one oral lesion such as dry mouth, bad taste in the mouth, degraded taste faculty, increased teeth decay rate, and gum bleeding. These problems affect the different aspects of QOL [12]. In general, physical and social performance disorders decrease the QOL of HD patients [13, 14].

The main objective of palliative care is to improve QOL. These interventions improve the quality of care and decrease medication expenses [10]. Treatment of ESRD patients is mostly of palliative nature and attempts to improve QOL in the patients [15–17]. Improvement of the QOL of dialysis patients should be an objective of treatment programs [18].

Group intervention in the form of group therapy is one of the least expensive and easy to access treatment and care methods for patients who deal with a wide range of problems and challenges, including problems in implementing disease coping methods and creating behavioral or life style changes [19]. Studies have shown that psychotherapy and non-pharmaceutical interventions are effective in adding meaning to life, creating goals in life, and improving the QOL of patients with CKD [20]. There is reliable evidence that group psychotherapy interventions are effective in improving QOL, decreasing mental pressure, improving coping skills, and decreasing the problems with symptoms and pain in ESRD patients [4, 21]. Nurses can improve the QOL of patients through primary intervention and group sessions to provide consultation and education services to ESRD and HD patients [22].

Given the fact that educational and psychotherapy interventions have positive effects on QOL and that the elements of QOL are important for CKD patients under HD, the present study is an attempt to determine the effects of supportive and educational group therapy on the QOL of HD patients.

## Methods

### Design

The study was carried out as a quasi-experimental study of HD patients living in Khorramabad City-Iran. The study took 10 months to complete, from June 2018 to April 2019.

### Participants

The study population consisted of all HD patients in Shohadaie Ashaier Hospital in Khorramabad City (*n* = 140). The subjects were selected through convenience sampling based on inclusion criteria and then allocated to experimental and control groups based on the days of HD (Sat, Mon, and Wed patients as the experimental group and Sun, Tuesday, and Thursday patients as the control group). The participants were allocated to the experimental and control groups, and measurements were done before, immediately after, and 1 month after the intervention.

Inclusion criteria were history of HD more than 6 months, no physical impairment (hearing loss or blindness), age range 18–65 years; desire to participate, reading and writing ability, no history of kidney transplantation, and not participating in similar classes. In addition, patients with diagnosed mental illness (according to the file information) were not included. Missing more than two sessions, reluctance to participate, and hospitalization during the study were the exclusion criteria.

The sample size was determined based on the formula for one quantitative variable and two groups (LOC = 95 (1-α); test power = 90% (1-β)). The rest of the parameters in the formula were based on [20], and the result was 21 as the minimum sample size. Eventually, 32 patients were selected for each group. Before the study, the experimental and control groups were compared in terms of confounding variables (e.g. sex, marital status, occupation, education, domicile, average monthly income, and history of hemodialysis).

**Fig. 1:**
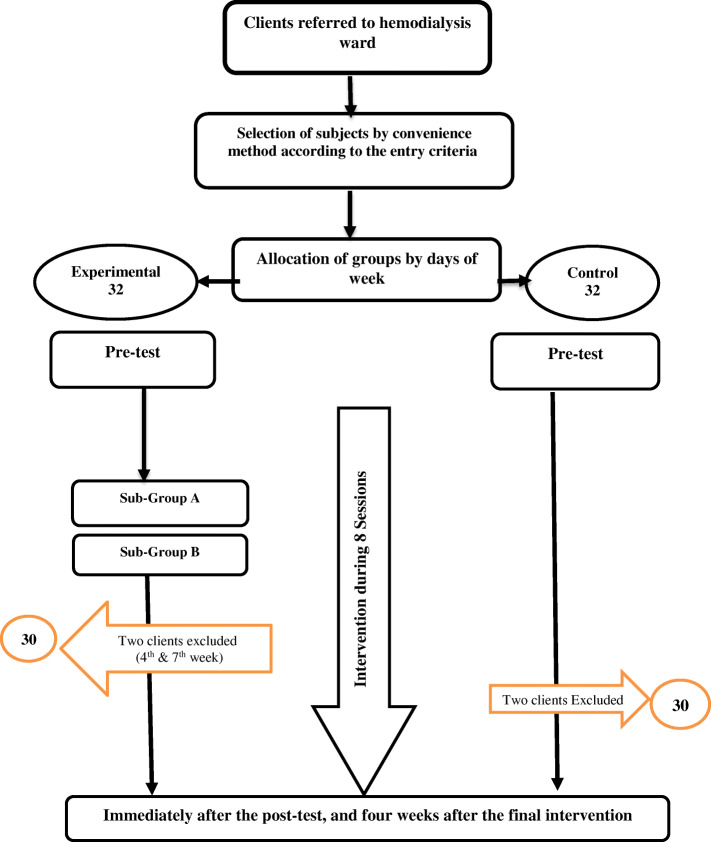
Study flow chart

After securing the required licenses, the researcher visited the research environment and briefed the officials and participants about the objectives and importance of the study. The participants signed a written letter of consent. The researcher tried to win the trust of the participants before asking them to fill out the questionnaire. According to the standards of group sessions and the number of patients, the participants of the experimental group were divided into two subgroups, A and B, each with 16 members. The experimental group received group therapy for eight sessions twice a week, each for 50mins. The sessions were held by the first author in the mosque of the hospital before the initiation of dialysis. Each session was held with the participation of 16 patients. Two patients left the study (one because of being hospitalized in the 3rd week and one because of travel and doing HD in another hospital in the 7th week, both in sub-group A of the experimental group). The interventions for subgroups A and B were quite similar. Patients in the control group received the routine dialysis interventions, which were the same for all patients including the participants in the experimental groups. In addition, two patients left the control group, so both groups completed the study with 30 participants each (Fig. 1). The participants filled out the questionnaires at the end of the last session and 1 month after the intervention.

#### Data gathering tool

A demographics form was designed by the researchers to cover the such information as age, sex, marital status, education, job, income, and dialysis time and period.

### Kidney disease quality of life short form (KDQOL-SFTM)

The KDQOL-SF™ was designed by Hays to assess the quality of life of patients. The questionnaire was developed in the US and has been translated into several languages. It contains 79 questions and measures the QOL of renal patients based on two sub-scales, a general health sub-scale (eight aspects) and a special sub-scale (ten aspects) [23, 24]. The questionnaire was tested and normalized in Iran with 212 renal patients. Table 1 lists the Cronbach’s alpha of the tool [24].

**Table 1:** KDQOL-SFTM and the subscales

KDQOL-SF™	Number of Items	Cronbach ‘s Alpha
**ESRD-targeted area**		
Symptom/Problem list(S/PL)	**12**	**0.92**
Effects of kidney disease (EKD)	**8**	**0.89**
Burden of kidney disease (BKD)	**4**	**0.86**
Work Status (WS)	**2**	**0.71**
Cognitive function (CF)	**3**	**0.74**
Quality of social interaction (QSI)	**3**	**0.77**
Sexual function (SexF)	**2**	**0.92**
Sleep (S)	**4**	**0.77**
Social support (SS)	**2**	**0.76**
Dialysis staff encouragement (DSE)	**2**	**0.81**
Patient satisfaction (PS)	**1**	
**36 items of health survey (SF-36)**		
Physical functioning (PF)	**10**	**0.93**
Role-Physical limitation (RPL)	**4**	**0.89**
Pain	**2**	**0.88**
General health (GH)	**5**	**0.74**
Emotional well-being (EW)	**5**	**0.73**
Role-emotional limitation (REL)	**3**	**0.79**
Social function (SocF)	**2**	**0.82**
Energy/fatigue (E/F)	**4**	**0.79**

### Sessions content

The session’s content was based on credible papers and sources [25–27] on the assessment of the QOL of HD patients. Before implementation, validity of the sources was checked using content validity and providing the sources to 10 experts. Cognitive counseling techniques including emotional venting, interpersonal learning, self-awareness, clarification, restatement, reflection, self-awareness, role playing, and rotational and purposeful questions were used.

#### Session one

Pretest, introduction and familiarization, clarification of the objectives of the sessions and the general structure, discussion of expectations, discussion of the role of self-care and self-awareness in the recovery process, and listening to the participants’ problems.

#### Session two

A lecture and group discussion about chronic renal failure, HD, life with HD, giving time to the patients to express their feelings, viewpoints, and concerns, then answering the patients’ questions during the group discussion.

Home assignment: each patient was asked to report their experience with general health problems.

#### Session three

Group discussion about the physical and mental side-effects of dialysis, prevention, and treatment, coping and attenuating the side-effects to preserve one’s social role, and giving feedback to patients.

Home assignment: To record concerns and problems about everyday activities and interactions with others.

#### Session four

Group discussion about the third session home assignment and emotional and sensory intervention. This session was aimed at educating the participants about relaxation methods along with positive mental image to decease probable tensions of the day.

#### Sessions five and six

Emotional and mental intervention; the main theme was to educate the participants about identifying their emotions and feelings and how to express them. In addition, the participants were encouraged to vent their suppressed emotions and feelings and in return the instructor and group members showed them sympathy and gave feedback. In addition, the importance of being independent, self-care, quality of social interactions, having a positive and good feeling, spiritual beliefs, the role of pain and agony in life, and the constructive effect of spiritual beliefs in alleviation of pains were emphasized to the participants.

#### Session seven

Spiritual interventions; the main theme of this session was care from a spiritual viewpoint. The role of pain and agony in life and its constructive effect and the development and expansion of ethical merits were examined. The relationships of man and nature, man and God, and man and the environment were also discussed.

#### Session eight

Summarizing, concluding, answering the participants’ questions, and giving a general review of the previous session. Before concluding the course, the authors showed their gratitude to the participants.

### Data analysis

Normal distribution of the questionnaire scores and other qualitative variables was checked using KS test. One-way ANOVA, independent t-test, Tukey ad-hoc test, and Mann Whitney U test were used depending on the variables and data distribution.

### Ethical consideration

All procedures were in accordance with the ethical standards of the institutional and national research committee and with the 1964 Helsinki declaration and its later amendments or comparable ethical standards. At the end of the study and given the effectiveness of group therapy, two intensive group therapy sessions were performed for the participants of the control group.

Kermanshah University of Medical Sciences approved the study under code No. 97146 and by the ethics committee under code No. IR.KUMS.REC.1397.043.

## Results

The study was completed with 60 patients, 88.33% men and 11.67% women. The mean age, number of children, and dialysis time duration of the experimental and control groups were 48.6 and 50.83 years, 2.1 and 2.2 children, and 3.7 and 3.26 years, respectively. Given the normal distribution of these three variables (K-S test), independent t-test did not show a significant difference between the two groups (*p* > 0.05). The rest of the demographical variables are listed in Table 2. Clearly, the two groups are identical in terms of sex, marital status, education, job, domicile, and average monthly income (*p* > 0.05).

**Table 2:** Demographic characteristics

Variables		Experimental N (%)	Control N (%)	*P*_value_
Age^a^	Less than 35 Y	1 (3.3)	1 (3.3)	0.317
	35–55 Y	22 (73.3)	18 (60)	
	More than 55 Y	7 (23.3)	11 (36.7)	
Sex^a^	Female	3 (10)	4 (13.3)	0.69
	Male	27 (90)	26 (86.7)	
Marital status	Unmarried	29 (96.7)	28 (93.3)	0.557
	Married	1 (3.3)	2 (6.6)	
Education	Elementary	21 (70)	25 (83.3)	0.301
	High school	6 (20)	3 (10)	
	Higher education	3 (10)	2 (6.7)	
Job	Employed	4 (13.3)	6 (20)	
	Unemployed	23 (76.6)	21 (70)	
	Housewife	3 (10)	3 (10)	
Monthly Income	Less than 800$	26 (86.7)	24 (80)	0.317
	800–1200$	1 (3.3)	5 (16.7)	
	More than 1200$	3 (10)	1 (3.3)	
Hemodialysis History	Less than 1 Y	4 (13.3)	4 (13.3)	0.92
	1–3 Y	13 (43.3)	17 (56.7)	
	More than 3Y	13 (43.3)	9 (30)	
Domicile	Urban	25 (83.3)	26 (86.7)	0.72
	Rural area	5 (16.7)	4 (13.3)	

^a^Yates correction test

With regard to the main variables, K-S test showed that the variables in the ESRD-targeted area, health survey, and KDQOL were of normal distribution in both groups and in the three stages of measurement (*p* > 0.05). Some of the sub-scales, such as symptom/problem list, social support, and social function, were of normal distribution in the experimental group only before intervention (*p* > 0.05).

**Fig. 2:**
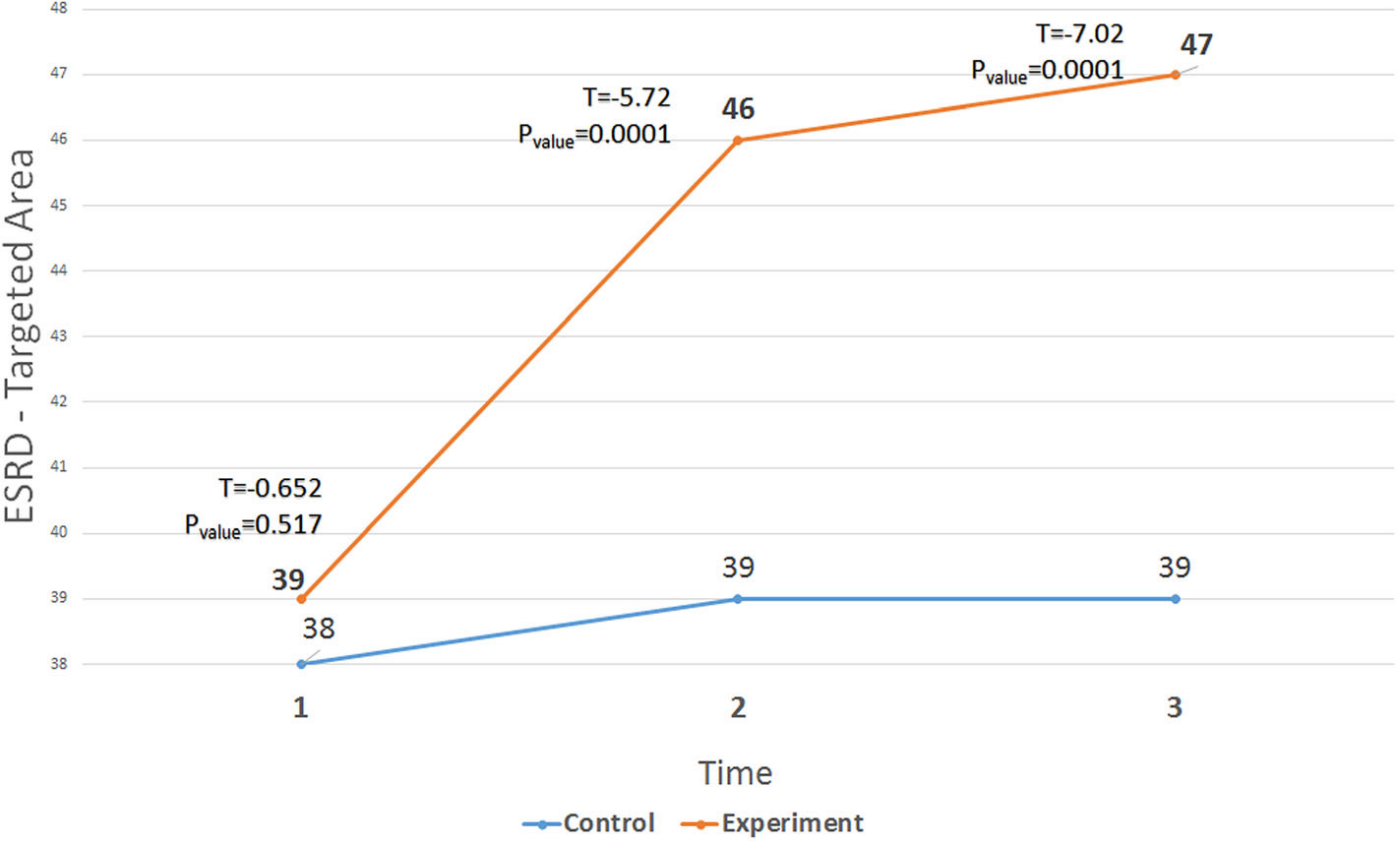
The trend of changes in the mean scores of ESRD-targeted areas in the three stages (before, immediately after and follow-up) in the two groups

**Fig. 3:**
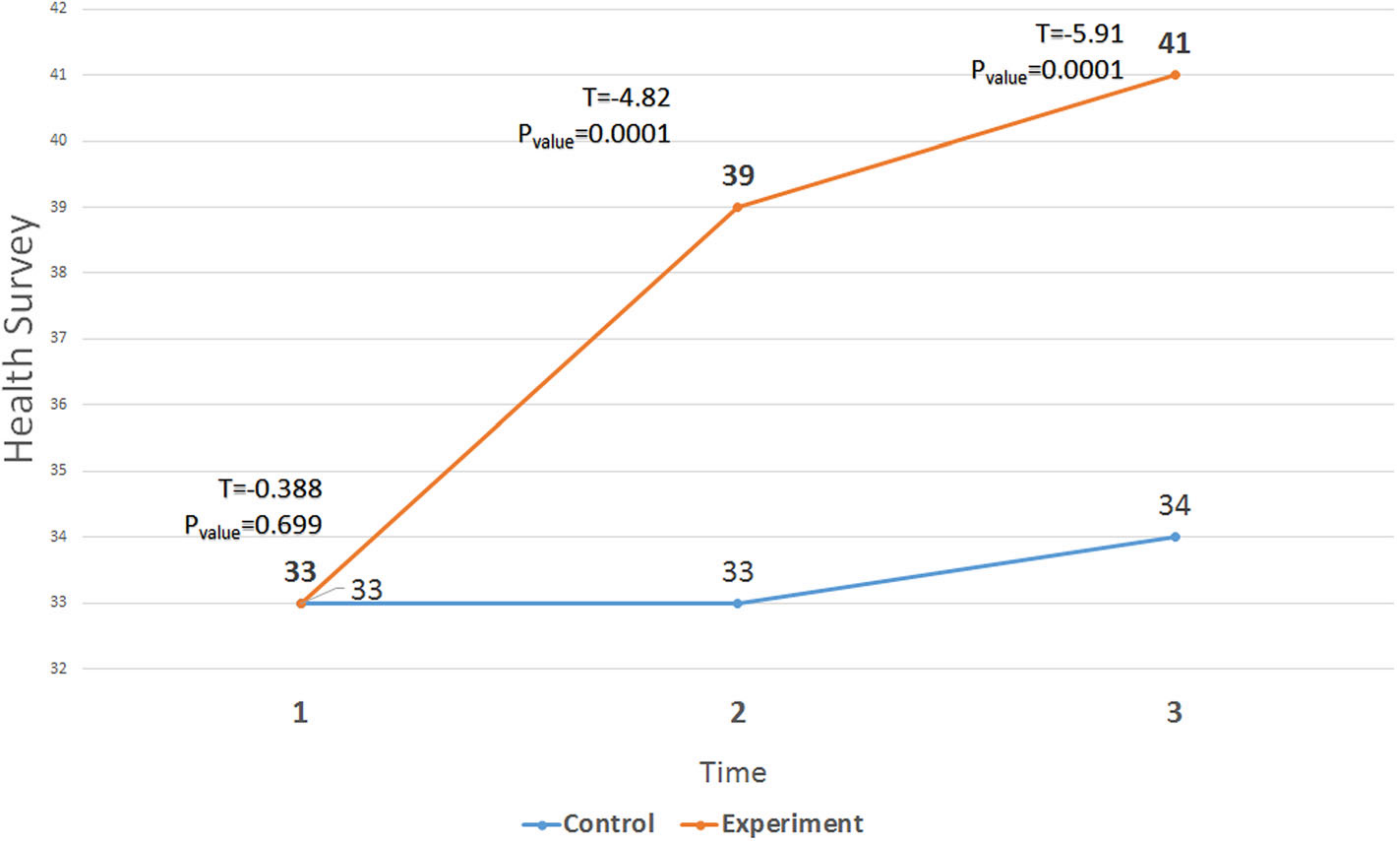
The trend of changes in the mean scores of Health survey in the three stages (before, immediately after and follow-up) in the two groups

**Fig. 4:**
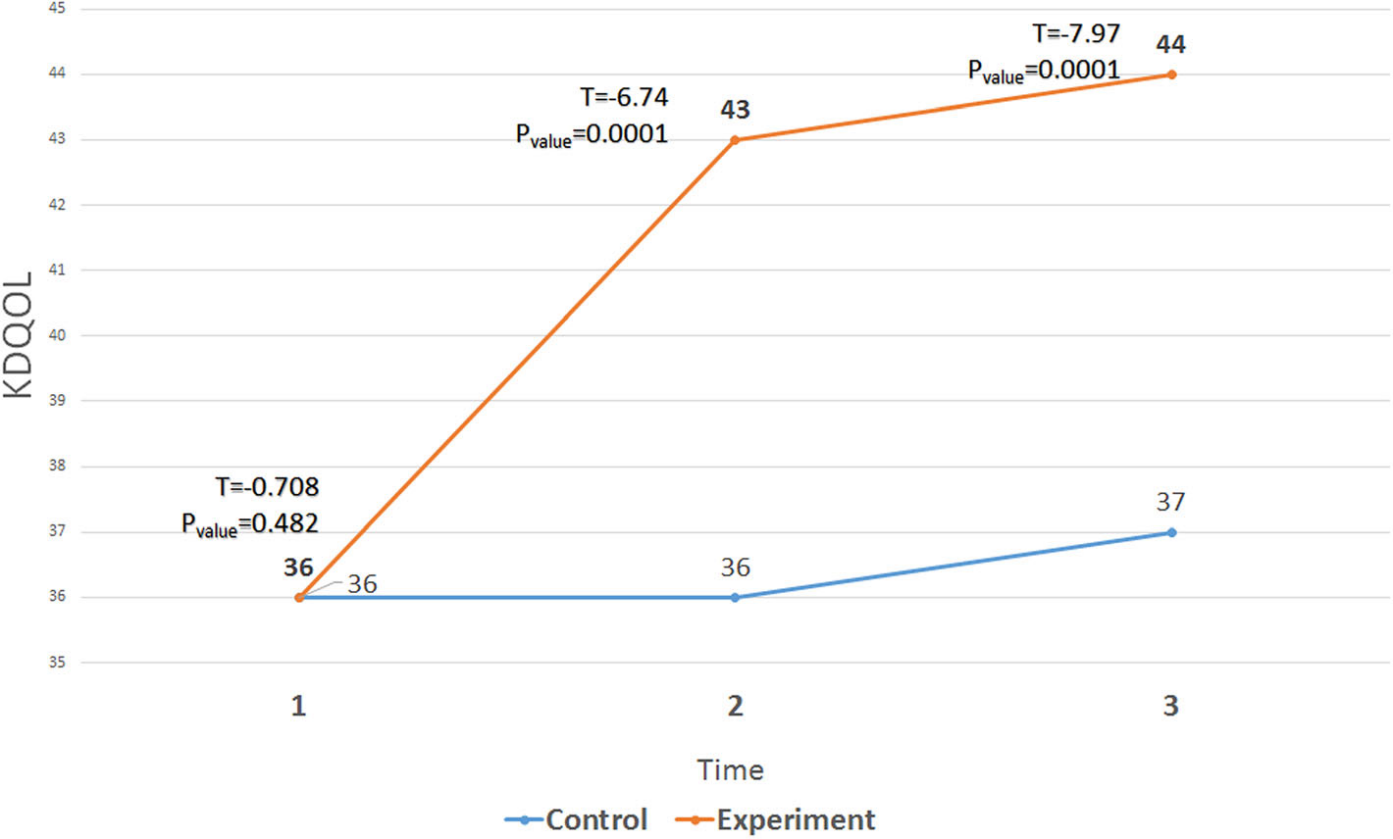
The trend of changes in the mean scores of KDQOL in the three stages (before, immediately after and follow-up) in the two groups

The ANOVA with repeated measures showed significant differences in the experimental group before, immediately after, and 1 month after the intervention in terms of the ESRD-targeted area (Fig. 2), health survey (Fig. 3), and KDQOL (Fig. 4). In addition, Tukey ad-hoc test showed a significant difference in terms of the mean score of these variables before, immediately after, and 1 month after the intervention in the experimental group. However, in terms of general health, there was a significant difference in the experimental group only before the intervention and immediately after the intervention (Table 3).

**Table 3:** Mean scores of KDQOL, ESRD-targeted area, and Health

	Time	Mean ± SD		T	*P*_Value_
		Control	Experimental		
ESRD-targeted area	Pre	38.57 ± 4.66	39.39 ± 3.9	−0.652	0.517
	Post	39.72 ± 4.27	46.32 ± 4.52	−5.72	0.0001
	Follow up	39.25 ± 4.32	47.57 ± 4.4	−7.02	0.0001
Health survey	Pre	33.39 ± 4.92	33.84 ± 3.96	−0.388	0.699
	Post	33.75 ± 5.36	39. 14 ± 2.95	−4.82	0.0001
	Follow up	34.14 ± 5.13	41.25 ± 4.13	−5.91	0.0001
KDQOL	Pre	36.39 ± 3.88	36.99 ± 2.65	−0.708	0.482
	Post	37.2 ± 4.03	43.3 ± 2.87	−6.74	0.0001
	Follow up	37.1 ± 4.08	44.9 ± 3.47	−7.97	0.0001

Independent t-test showed that there was no significant difference between the two groups in terms of all the aspects of QOL before the intervention. However, there was a significant difference immediately after and during the follow-up period of the intervention in terms of the ESRD-targeted area, health survey, KDQOL and some sub-scales (*p* < 0.05). The variables that were not significantly different were symptom/problem list, burden of kidney disease, work status, quality of social interaction, emotional well-being, and social function (*P* > 0.05) (Table 4).

**Table 4:** Mean sub-scales scores of KDQOL

		Mean ± SD			P**_Value_
	Group	Pre	Post	Follow up	
Symptom/Problem list(S/PL)	Control	40.08 ± 7.84	40.67 ± 10.48	41.33 ± 11.14	0.819
	Experimental	39.85 ± 7.57	46.83 ± 14.31	47.62 ± 14.86	0.031
	P*_value_	0.798	0.118	0.122	
Effects of kidney disease (EKD)	Control	29.27 ± 9.62	29.27 ± 9.27	28.85 ± 9.67	0.549
	Experimental	31.3 ± 6.41	40.41 ± 12.38	41.77 ± 11. 6	0.0001
	P*_value_	0.34	0.0001	0.0001	
Burden of kidney disease (BKD)	Control	32.71 ± 14.92	34.58 ± 14.93	33.96 ± 16.06	0.247
	Experimental	35.83 ± 10.12	41.95 ± 10.41	43.23 ± 10.59	0.012
	P*_value_	0.405	0.124	0.708	
Work Status (WS)	Control	18.33 ± 21.71	22.5 ± 22.12	22.5 ± 23.07	0.016
	Experimental	18.33 ± 15.99	25 ± 18.57	25 ± 17.37	0.066
	P*_value_	0.749	0.605	0.588	
Cognitive function (CF)	Control	45.55 ± 16.16	49.55 ± 16.32	47.8 ± 15.16	0.006
	Experimental	46.03 ± 10.72	56.45 ± 8.01	56.13 ± 8.28	0.0001
	P*_value_	0.952	0.137	0.026	
Quality of social interaction (QSI)	Control	60.44 ± 18.69	59.77 ± 19.85	57.33 ± 19.62	0.368
	Experimental	60.54 ± 17.75	63.43 ± 15.62	61.04 ± 17.76	0.64
	P*_value_	0.782	0.81	0.77	
Sexual function (SexF)	Control	41 ± 11.25	40.83 ± 10.99	40.83 ± 10.99	0.368
	Experimental	40.42 ± 6.2	44.08 ± 7.58	44.5 ± 6.1	0.0001
	P*_value_	0.643	0.066	0.046	
Sleep (S)	Control	49.17 ± 14.15	50 ± 13.34	47.83 ± 15.63	0.016
	Experimental	47.13 ± 12.43	53.67 ± 12.65	59.13 ± 12.56	0.0001
	P*_value_	0.051	0.056	0.004	
Social support (SS)	Control	30.72 ± 12.8	31 ± 11.4	31.72 ± 12.27	0.488
	Experimental	31.19 ± 6.42	36.88 ± 6.29	39.91 ± 7.6	0.0001
	P*_value_	0.546	0.005	0.003	
Dialysis staff encouragement (DSE)	Control	37.96 ± 8.93	40.31 ± 9.5	41.14 ± 9.09	0.018
	Experimental	40.37 ± 11.07	45.93 ± 8.13	48.56 ± 6.94	0.0001
	P*_value_	0.881	0.003	0.0001	
Patient satisfaction (PS)	Control	39 ± 22.07	41 ± 13.48	39.17 ± 10.35	0.379
	Experimental	41.21 ± 17.08	54.87 ± 17.08	56.09 ± 18.82	0.0001
	P*_value_	0.453	0.002	0.0001	
Physical functioning (PF)	Control	38.17 ± 10.46	37.67 ± 11.65	36.5 ± 10.18	0.247
	Experimental	39.5 ± 11.09	41.67 ± 10.69	43.81 ± 9.71	0.003
	P*_value_	0.317	0.029	0.003	
Role-Physical limitation (RPL)	Control	21.67 ± 25.2	23.33 ± 25.37	21.67 ± 25.2	0.368
	Experiment	20.83 ± 13.27	29.17 ± 11.53	31.67 ± 18.49	0.014
	P*_value_	0.543	0.085	0.04	
Pain (P)	Control	31.66 ± 10.24	30.42 ± 10.21	31.75 ± 10.57	0.867
	Experimental	31.75 ± 9.76	36.33 ± 9.3	35.75 ± 9.19	0.006
	P*_value_	0.782	0.033	0.154	
General health (GH)	Control	39 ± 12.06	37.67 ± 12.44	37.5 ± 12.3	0.368
	Experimental	42.61 ± 7.12	46.19 ± 4.27	51.69 ± 5.12	0.0001
	P*_value_	0.635	0.041	0.0001	
Emotional well-being (EW)	Control	40.4 ± 19.05	42.17 ± 19.76	43.8 ± 21.05	0.004
	Experimental	38.87 ± 5.82	47.33 ± 3.94	49.15 ± 6.33	0.0001
	P*_value_	0.995	0.282	0.35	
Role-emotional limitation (REL)	Control	21.10 ± 18.53	22.22 ± 23.7	21.22 ± 22.19	0.931
	Experimental	22.22 ± 15.98	23.33 ± 17.83	25.55 ± 14.34	0.81
	P*_value_	0.724	0.643	0.306	
Social function (SocF)	Control	38.6 ± 4.63	39.66 ± 4.35	42.81 ± 6.08	0.007
	Experimental	37.55 ± 5.21	43.38 ± 9.51	47.54 ± 8.67	0.0001
	P*_value_	0.089	0.003	0.006	
Energy/fatigue (E/F)	Control	36.53 ± 9.1	39.87 ± 7.94	37.87 ± 9.47	0.521
	Experimental	38.4 ± 8.81	45.73 ± 9.88	44.83 ± 10.10	0.0001
	P*_value_	0.509	0.0001	0.007	

* U-Mann-Whitney test **Friedman test

## Discussion

After the group therapy, the quality of life of the HD patients was significantly improved compared to that before the intervention and the control group. The increase can be attributed to the intervention. There are several research reports on the effect of educational intervention and counseling on the QOL of HD patients [21, 28, 29]. These studies confirm the effects of education and counseling about life style, drugs, diet, and concerns and problems of these patients on their QOL [4, 10, 30]. In addition, participation in a group and the support that these patients can provide to each other alleviates their stress and improves their life expectancy, which in return improves their QOL [11, 20, 31, 32]. The QOL of HD patients is lower than that of normal individuals [26]. One of the least expensive and easiest therapeutic and care methods for patients who deal with a wide range of problems and challenges, including problems coping disease with and creating behavioral changes in life style, is group interventions [33].

In terms of the sub-scales of QOL, the results showed that the SQI score after the intervention was significantly different between the two groups. In terms of social interaction, the intervention had a positive effect. In addition, the group therapy intervention improved GH scores significantly. In terms of EW, the intervention was significantly effective and improved it compared to that before the intervention. There was a significant increase in SoF score in the intervention group compared to the control group. In addition, the mean scores of EKD, BKD, QSI, EW, and ERL due to emotional problems improved significantly after the intervention in the experimental group. Other studies have also reported improvement in most of these variables, although they have used different questionnaires [20, 30].

Moreover, the intervention affected SS and WS, but the effect was not significant. The findings are consistent with [34]. The fact that the disease is chronic and debilitating and takes a lot of time and energy of the patient may explain this. Therefore, the intervention was ineffective in this regard and there was a need for support by organizations other than health organizations.

The results showed that the educational and supportive group intervention created no positive change in terms of sleep performance. Similar results were reported by Bayoumi et al. (2015), thus there is a need for more studies on the sleep of these patients [35].

### Limitations

In terms of environmental limitations, nature of the disease, and mental status of patients, the mental and spiritual condition of the subjects affected their answers to the questionnaire. The author did not have any way to control this. In terms of the advantages of the study, using a special quality of life tool and group therapy intervention are notable. Female patients were not interested in participating in the study with the efforts made so, unfortunately, only a small number enrolled. Moreover, due to the possibility of contact between patients during dialysis sessions and hospital stays, instead of random allocation of the participants into control and experimental groups the groups were based on dialysis days to minimize the possibility of contact between the participants of the two groups and to minimize empirical and information exchange between them.

## Conclusion

The group therapy intervention with an educational and supportive approach had a significant effect on special and general health aspects and the QOL of the patients in the intervention group. Implementation of such intervention can improve the QOL of HD patients.

## Data Availability

The datasets used and analyzed during the current study are available from the corresponding author on reasonable request.

## Abbreviations

QOL: Quality of life
ESRD: End- Stage Renal Disease
CKD: Chronic Kidney Disease
KUMS: Kermanshah University of Medical Sciences
KDQOL-SFTM: Kidney Disease Quality of Life Short Form
HD: Hemodialysis
S/PL: Symptom/Problem list
EKD: Effects of kidney disease
BKD: Burden of kidney disease
WS: Work Status
CF: Cognitive function
QSI: Quality of social interaction
SexF: Sexual function
SS: Social support
DSE: Dialysis staff encouragement
PS: Patient satisfaction
PF: Physical functioning
RPL: Role-Physical limitation
GH: General health
EW: Emotional well-being
REL: Role-emotional limitation
SocF: Social function
E/F: Energy/fatigue
